# Supplementary material for: Association between Dietary Habits, Food Attitudes, and Food Security Status of US Adults since March 2020: A Cross-Sectional Online Study
Source: Nutrients. 2022 Nov 3;14(21):4636. doi: 10.3390/nu14214636 (PMC9658187; doi:10.3390/nu14214636)
Supplement: Supplementary file 1 [file nutrients-14-04636-s001.zip › nutrients-1988096-supplementary.pdf]

Supplementary Table S1. US Adults' Dietary habits, Food Attitudes and Food security status since March 2020

Dietary Habits: For the following foods listed, indicate if the amount you have consumed has increased, decreased, remained the same, or never consumed since March 2020

Food Category/ Item	Increased	Decreased	No change	Never consumed pre-COVID
Milk and non-milk alternatives, yogurt and cheese				
Margarine or butter				
Fruit (fresh, frozen or canned)				
Fruit juice				
Non-starchy vegetables such as broccoli, carrots, green beans (fresh, frozen or canned), salad				
Vegetable or tomato juice				
Eggs, chicken, turkey				
Beef, pork or lamb				
Processed meats such as bacon, hot dogs, sausage, salami, bologna or luncheon meats				
Fish and shellfish				
Cold breakfast cereal				
White bread including pita bread				
Dark bread including pita bread				
French fried potatoes				
Starchy vegetables such as corn, peas, and beans				
White rice or pasta				
Brown rice or whole-grain pasta				
Potato chips or other salty snacks such as crackers				
Nuts or seeds				
Peanut butter or other nut butter spreads				
Sweets – candy, cake, cookies, pies				
Oil such as olive, sunflower				
Water				
Coffee or tea				
Immune enhancing beverages – such as ginger, curcumin				
Beer or wine				
Hard liquor – such as whiskey, rum, vodka, gin				
Low calorie carbonated beverages such as diet colas				
Carbonated beverages with sugar such as colas, lemonade, fruit drinks				

Food Attitudes: Since March 2020, indicate if your attitude has changed for the following statements:

	Increased	Decreased	No change	Never had these thoughts pre-COVID
I find that when I start eating certain foods, I end up eating much more than planned				
I find myself continuing to consume certain foods even though I am no longer hungry				
I eat to the point where I feel physically ill				
I spend a lot of time feeling sluggish or fatigued from overeating				
I find myself constantly eating certain foods throughout the day				
My behavior with respect to food and eating causes significant distress.				

What is your current height in feet and inches? For example, 5 feet 8 inches

What is your current weight in pounds?

Since March 2020, has your weight:

a. Increased
b. Decreased
c. No change

Which of the following conditions do you have? (select all that apply)

◎ Cancer
◎ Dementia / Alzheimer's
◎ Depression
◎ Diabetes (high blood sugar)
◎ Diverticulosis/Diverticulitis
◎ Gastric reflux
◎ Heart disease (includes high blood pressure, heart attack, artery disease, stroke, angina)
◎ Irritable Bowel
◎ Liver disease (cirrhosis, fatty liver)
◎ Lung disease
◎ Nausea/Vomiting
◎ Other (please indicate) _______________
◎ None of the above

Since March 2020, have you tried a diet?

a. Yes
b. No

If yes, which of the following diets have you tried/currently on:

a. Low carbohydrate diet
b. Low fat diet
c. Low salt diet
d. Plant-based diet
e. Weight management diet
f. Other:

Since March 2020, have you begun taking nutritional supplements?
a. Yes
b. No

If yes, which of the following nutritional supplements are you currently taking?
a. Calcium
b. Magnesium
c. Multi-vitamin
d. Iron
e. Omega 3
f. Omega 6
g. Protein (bars, shakes, powder)
h. Vitamin B complex
i. Vitamin C
j. Vitamin D
k. Other:

Since March 2020, you are currently staying at home _____ of the time:
a. 25% or less
b. 50-75%
c. 75-95%
d. Never have left my house

Since March 2020, has your physical activity:
a. Increased
b. Decreased
c. No change

Lifestyle Habits: For the following statements, indicate which activities have increased, decreased, stayed the same, or never did since March 2020:

	Increased	Decreased	Stayed the same	Never did pre-COVID
Dining at restaurants, cafes, etc				
Preparing/cooking meals in the home				
Meal kit services (e.g., Blue Apron, Hello Fresh)				
Take-out/delivering of meals from restaurants, cafes, etc				
Grocery shopping in the store				
Grocery shopping online				
Reading/Studying				

Sleep hours and quality				
Smoking (Cigarettes, Cigar, Hookah)				
Socializing outside the home				
Use of electronic devices				

Food Security: For the following 6 statements, click the best answer since March 2020:
The food that (I/we) bought just didn't last, and (I/we) didn't have money to get more
[ ] Often true
[ ] Sometimes true
[ ] Never true
[ ] Don't know or Refused to answer

(I/we) couldn't afford to eat balanced meals.
[ ] Often true
[ ] Sometimes true
[ ] Never true
[ ] Don't know or Refused to answer

Did (you/or other adults in your household) ever cut the size of your meals or skip meals because there wasn't enough money for food?
[ ] Yes
[ ] No
[ ] Don't know

How often since March 2020, has this happen?
[ ] The past 2 months
[ ] The past month
[ ] Only once
[ ] Don't know

Since March 2020, did you ever eat less than you felt you should because there wasn't enough money for food?
[ ] Yes
[ ] No
[ ] Don't Know

Since March 2020, were you every hungry but didn't eat because there wasn't enough money for food?
[ ] Yes
[ ] No
[ ] Don't Know

Demographics
What is your age range?

a. 18-24 years old
b. 25–29 years old
c. 30-49 years old
d. 50-59 years old
e. 60-69 years old

f. 70 years old or older

What is your sex?

a. Male
b. Female
c. Other

What is your race/ethnicity?

a. African American
b. Asian
c. Caucasian
d. Hispanic
e. Native American
f. Other: ______________

What region of the United States do you reside for at least 8 months of the year?

a. New England (Connecticut, Maine, Massachusetts, Rhode Island, Vermont)
b. Mid-Atlantic (New Jersey, New York, Pennsylvania)
c. South Atlantic (Delaware, Florida, Georgia, Maryland, North Carolina, South Carolina, Virginia, Washington DC, West Virginia)
d. East North Central (Illinois, Indiana, Michigan, Ohio, Wisconsin)
e. East South Central (Alabama, Kentucky, Mississippi, Tennessee)
f. West North Central (Iowa, Kansas, Minnesota, Missouri, Nebraska, North Dakota, South Dakota)
g. West South Central (Arkansas, Louisiana, Texas)
h. Mountain (Arizona, Colorado, Idaho, Montana, Nevada, New Mexico, Utah, Wyoming)
i. Pacific (Alaska, California, Hawaii, Oregon, Washington)

What is your education level?

a. No schooling completed
b. Nursery school to 8th grade
c. Some high school, no diploma
d. High school graduate, diploma or the equivalent (for example: GED)
e. Some college credit, no degree
f. Trade/technical/vocational training
g. Associate degree
h. Bachelor's degree
i. Master's degree
j. Professional degree
k. Doctorate degree

What is your current employment status?

a. Full-time
b. Part-time
c. Unemployed
d. Other

What is your marital status?

a. Married

b. Single
c. Widowed
d. Divorced
e. Other

Besides yourself, how many people live in the household?

____
